# Retinal status analysis method based on feature extraction and quantitative grading in OCT images

**DOI:** 10.1186/s12938-016-0206-x

**Published:** 2016-07-22

**Authors:** Dongmei Fu, Hejun Tong, Shuang Zheng, Ling Luo, Fulin Gao, Jiri Minar

**Affiliations:** 1 School of Automation and Electrical Engineering, University of Science and Technology Beijing, Xueyuan Road 30, Haidian District, Beijing, China; 2 The 306th Hospital of People’s Liberation Army, Beijing, China; 3 Dept. of Telecommunications, Faculty of Electrical Engineering and Communication, Brno University of Technology, Czech, Brno, Czech Republic

**Keywords:** Retinal OCT images, Image processing, Morphological characterization, Feature quantification, Grade evaluation

## Abstract

**Background:**

Optical coherence tomography (OCT) is widely used in ophthalmology for viewing the morphology of the retina, which is important for disease detection and assessing therapeutic effect. The diagnosis of retinal diseases is based primarily on the subjective analysis of OCT images by trained ophthalmologists. This paper describes an OCT images automatic analysis method for computer-aided disease diagnosis and it is a critical part of the eye fundus diagnosis.

**Methods:**

This study analyzed 300 OCT images acquired by Optovue Avanti RTVue XR (Optovue Corp., Fremont, CA). Firstly, the normal retinal reference model based on retinal boundaries was presented. Subsequently, two kinds of quantitative methods based on geometric features and morphological features were proposed. This paper put forward a retinal abnormal grading decision-making method which was used in actual analysis and evaluation of multiple OCT images.

**Results:**

This paper showed detailed analysis process by four retinal OCT images with different abnormal degrees. The final grading results verified that the analysis method can distinguish abnormal severity and lesion regions. This paper presented the simulation of the 150 test images, where the results of analysis of retinal status showed that the sensitivity was 0.94 and specificity was 0.92.The proposed method can speed up diagnostic process and objectively evaluate the retinal status.

**Conclusions:**

This paper aims on studies of retinal status automatic analysis method based on feature extraction and quantitative grading in OCT images. The proposed method can obtain the parameters and the features that are associated with retinal morphology. Quantitative analysis and evaluation of these features are combined with reference model which can realize the target image abnormal judgment and provide a reference for disease diagnosis.

## Background

Optical coherence tomography (OCT) is an in vivo imaging technique that could rapidly acquire high resolution cross-section images of biological tissues microstructure [1]. The most significant medical contribution of OCT is the ophthalmology area, as it could provide the retinal structure and functional images that no other noninvasive diagnosis method can perform. Several medical researchers used OCT to acquire retinal statistical characteristics and analyzed different kinds of fundus diseases, such as macular oedema caused by diabetic retinopathy [2], drusen and drusenoid pigment epithelium detachment caused by non-neovascular age-related macular degeneration [3], X-linked retinoschisis [4], epiretinal membrane, macular hole, central serous chorioretinopathy [5] et al. In addition, there are researches on statistical analysis of normal retinal thickness [6].

Ophthalmologists diagnose the fundus diseases by analyzing the change of retinal features in OCT images; however, OCT instruments can produce large amounts of data in a short time, so it is difficult to analyze all data by manual method. In most cases, only a small number of selected images are analyzed, and this could cause the wasting of medical resources. Furthermore, manual analysis results are very depending on ophthalmologist’s personal experience and there is also lack of uniform quantization and evaluation standards. Therefore, rapid, accurate, objective detection and quantification of retinal features is the key of medical OCT images research and diagnosis of ophthalmic diseases, and this study has important theoretical significance and practical value.

In order to achieve the retinal features detection and quantification, computer image processing and analysis technologies have been widely applied in the field of medical OCT images. Quellec et al. [7] realized the automated identification of macular fluid-filled regions by analysis of retinal layer texture. Gregori et al. [8], Iwama et al. [9] and Chen et al. [10] utilized different methods to segment retinal drusen and made the different levels quantification and evaluation. The upper studies are aimed at the known cases of illness, and extract the pathological areas in image. Liu et al. [11, 12] utilized retinal geometry, texture, shape features to identify the presence of normal macula and each of three types of macular pathologies, but without the analysis of pathological severity degree. Koprowski et al. [13] described a method for automatic analysis of selected choroidal diseases by features definition and quantification. By using the feature data acquired from OCT, Koprowski et al. [14] also proposed an automatic method for the analysis of OCT images in assessing the severity degree of glaucoma. Xu et al. [15] segmented the nerve fiber layer and analyzed OCT data for glaucoma detection. These studies identified or classified the limited certain kinds of diseases according to the extracted pathologic features.

The upper studies are either for the extraction of pathological area in known cases of illness or for the features extraction and classification of limited certain kinds of diseases. In practical diagnostic process, the obtained retinal images are usually complex and the abnormal categories would not be limited within the scope of certain kinds of known cases. Furthermore, retinopathy is gradually evolved and the abnormal early detection and early diagnosis has great significance. Therefore, in order to realize computer automatic analysis of retinal status, it is also needed to rely on the ophthalmologists’ practical diagnosis process to deal with different situations in reality.

When ophthalmologists interpret retinal OCT images, they focus on the locations of the lesions occur (macular areas, internal limiting membrane, retinal pigment epithelium, nerve fiber layer, etc) and the feature morphologies (retinal thickness, overall shape change, boundary smoothness, boundary continuity, etc) that are conducive to abnormal judgment. And they compare the specific tissue structure morphology with the known normal morphology. In the process of comparison, ophthalmologists do some extensive quantitatively analysis of the differences in morphology, such as retinal thickness (ratio) change, the morphological change of quantity, etc, and judge abnormal severity and lesion location. Finally ophthalmologists make the association between these morphological differences and corresponding disease categories, and give the diagnosis decision.

**Fig. 1:**
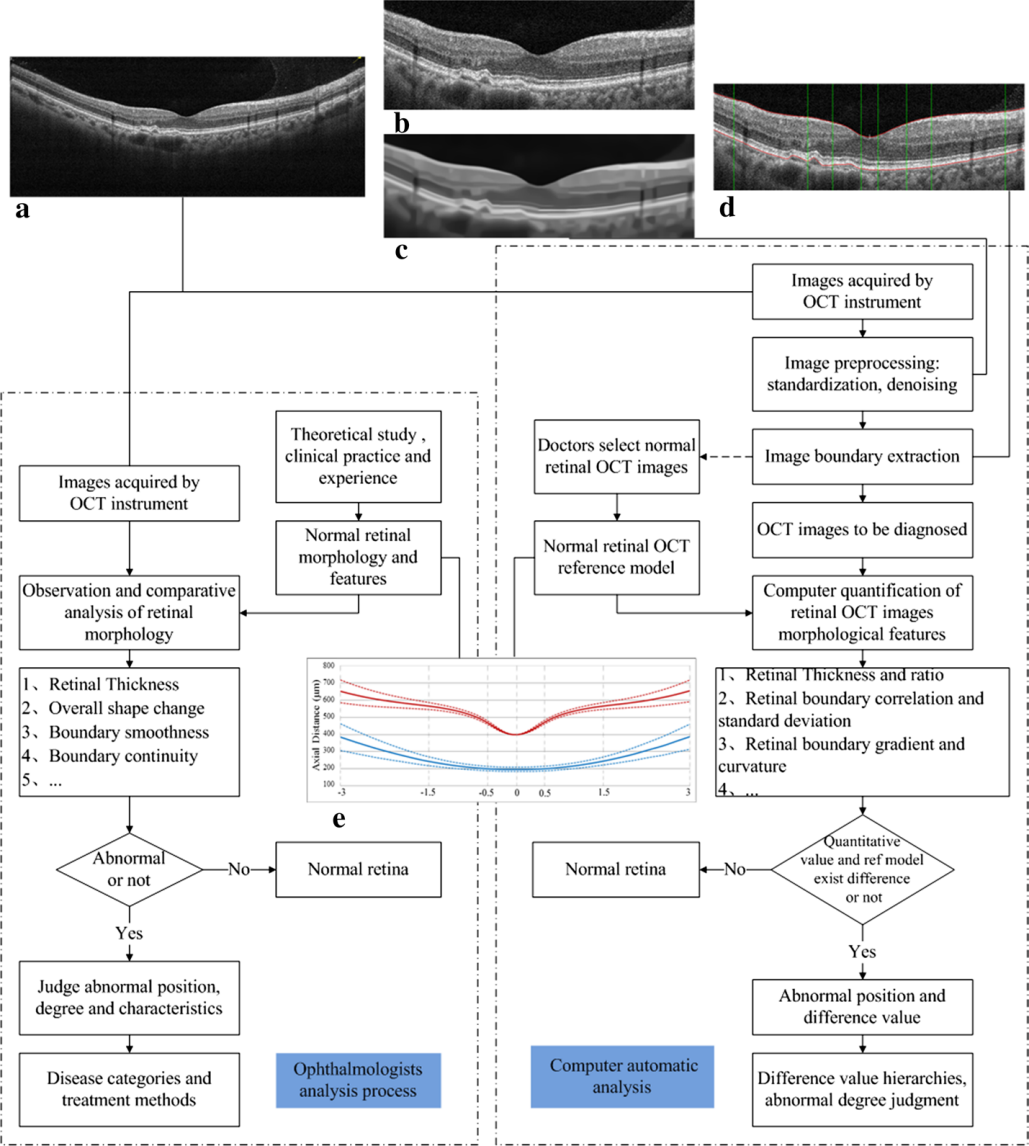
The contrast between ophthalmologist’s analysis process and computer automatic analysis process. The ophthalmologist’s practical diagnosis process is denoted with the *dotted box* on the *left side* and the *dotted box* on the *right side* shows the proposed retinal status computer automatic analysis process. **a** Original image, **b** image after standardization, **c** image de-noising results, **d** image boundary extraction results, **e** normal retinal OCT reference model

Based on the process of ophthalmologists’ observation and analysis on fundus retinal morphology in OCT images, this paper computerized the analysis and decision-making process, and put forward a retinal status automatic analysis method. The ophthalmologists’ analysis process and the proposed retinal status automatic analysis process diagram are shown in Fig. 1. In this paper, a normal retinal reference model is constructed to analogy the normal tissue structures that ophthalmologists are familiar with. Image processing methods such as boundary extraction, morphological characterization and features quantification are used to express of the ophthalmologists’ diagnosis process. Finally, evaluation of the results are presented by the way of abnormal grading.

## Material

This study analyzed 300 retinal OCT images, including 200 images judged as normal by ophthalmologists and the remaining 100 images with various abnormalities, such as drusen, macular epiretinal membrane, macular edema and macular hole, etc. These images are from 300 participants, aged from 18 to 78 years and they are acquired using Optovue Avanti RTVue XR (Optovue Corp., Fremont, CA) from the 306th Hospital of People’s Liberation Army, Department of ophthalmology. This study is supported by the National Natural Science Foundation of China. Image analysis was carried out in Matlab.

**Fig. 2:**
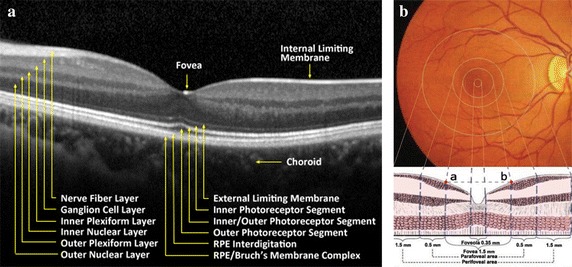
Diagram of normal retinal structure. **a** Normal retinal tissue layers image shot by OCT instrument. **b** Macular structure, including foveola, fovea, parafovea, perifovea regions

Retina is a transparent film layer located in the inner wall of eye. At present, the most advanced OCT instrument can distinguish 12 normal retinal tissue layers, as shown in Fig. 2a. In the posterior pole of the retina, there is a funnel-shaped depression pale, and that is the optical center of eye, known as the macular region, as shown in Fig. 2b. The structure and physiological activity of retina in this region are special, and it is easy to be affected by internal and external pathogenic factors. Therefore, this paper focuses on the morphological changes of retina in macular region. Retinal macula can be subdivided into three anatomical zones: (1) fovea, the center of macular region, and it is about 1.5 mm in diameter, that is, a optic disc diameter. The center of fovea is called foveola and it is about 0.35 mm in diameter. (2) parafovea, a circular ring area about 0.5 mm outside the fovea, and it contains ganglion cells, inner nuclear layer and outer plexiform layer called Henle fiber. (3) perifovea, a circular ring area about 1.5 mm outside the parafovea.

## Methods

### Image preprocessing and normal retinal reference model

#### Image standardization and de-noising

In the process of imaging, the OCT instrument auto focuses according to the size of the targets’ shape, changes the axial resolution of the image, and the retina appears with different degree of tilt in the image. So first of all, OCT images should be standardized. The aims of image standardization are: (1) unify the image size and axial resolution; (2) focus on macular central area; (3) make the whole retina in a horizontal position, in order to ensure the unity of the follow-up feature extraction.

The typical retinal OCT image has these characteristics: edge unclear, fuzzy and low signal-to-noise ratio, and thus de-noising is needed in preprocessing. The de-noising methods in OCT images mainly include complex domain methods [16, 17] and magnitude domain methods, while magnitude domain methods are more widely used and these methods may be studied in raw images domain [18–23] and sparse representation [24–26].

Five de-noising methods are compared in this paper, including Bayesian least squares–Gaussian scale mixture approaches (BLS-GSM) [18], non-local means (NL Means) [19], nonlinear complex diffusion filter (NCDF)[20], complex wavelet based dictionary learning methods (CWDL) [24] and Block-matching and 3D filtering (BM3D) [21].

Contrast-to-noise ratio (CNR), texture preservation (TP), edge preservation (EP), and equivalent number of looks (ENL) were computed for each de-noising method to compare the performance of the de-noising algorithms. The mentioned values are computed based on methods discussed in [27]. Table 1 shows the value of the measurements for each de-noising method of 20 OCT images, and the overall effect of BM3D algorithm is better than others. So BM3D algorithm was utilized in this paper for OCT image de-noising processing.

**Table 1:** Value of the measurements for each de-noising method

	BLS-GSM	NL means	NCDF	CWDL	BM3D
CNR	14.05 ± 4.46	17.48 ± 3.45	19.34 ± 5.32	25.68 ± 5.95	31.79 ± 8.38
EP	0.88 ± 0.04	0.96 ± 0.04	0.77 ± 0.05	0.74 ± 0.08	0.75 ± 0.09
TP	0.58 ± 0.11	0.41 ± 0.29	0.19 ± 0.06	0.07 ± 0.05	0.03 ± 0.04
ENL	35.89 ± 14.79	283.53 ± 116.55	12.9 ± 4.87	86.62 ± 25.27	510.94 ± 402.32

#### Normal retinal reference model

In clinical practice, ophthalmologists compare the feature and morphology differences between specific targets and ’standard’, and take the differences as symptom information. This paper built normal retinal reference model as ’standard’ to compare differences in identifying specific targets and it is conducive to obtain quantitative features for medical diagnosis.

When the macular lesions occur, internal limiting membrane (ILM) and retinal pigment epithelium (RPE) have morphological variation, and that is an important criteria of illness judgment. ILM is the inner boundary membrane of retina and RPE lower boundary is the outer boundary membrane of retina. Retina is defined between these two boundary membranes in medicine and some OCT instruments also take the detection data between these two membranes as retinal thickness data. So this paper chose ILM and RPE lower boundary to build the normal retinal reference model.

**Fig. 3:**
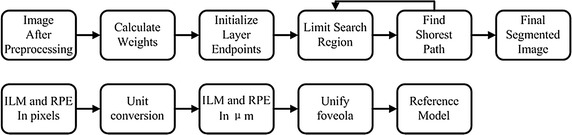
The generalized layer segmentation algorithm schematic and the construction of reference model. The segmentation algorithm was based on graph theory and dynamic programming and the segmentation of retinal layer was achieved by finding the shortest path in the limited search region. The first segmented boundaries were in pixels and the unit was converted according to the axial resolution. The foveola locations in all images were unified and the averaged boundaries were reference model

**Fig. 4:**
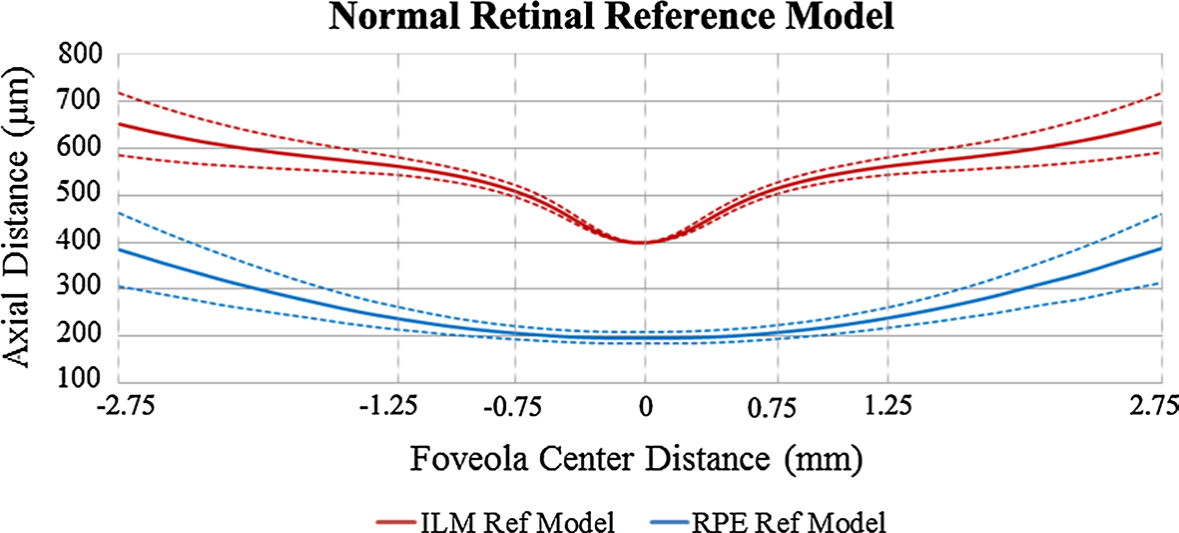
Normal retinal reference model. The solid lines are the average boundaries of normal retinal ILM and RPE, as the normal retinal reference model. The *dotted lines* are the variation range based on reference model standard deviation. The *horizontal axis* shows the distance to foveola center, and the numerical value is consistent with the Fig. 2b. The *vertical axis* represents the axial distance, and the retina thickness changes around the macular regions can be seen

This paper randomly selected 100 images from the 200 normal OCT images as the data base of references’ models and the other 100 images are used to test the effectiveness of the reference model. ILM and RPE boundary extraction is the foundation for retinal reference model construction. There are many studies on retinal layers segmentation [28–34], and this paper used the relatively stable extraction method based on graph theory and dynamic programming from literature [28]. The generalized schematic of the layer segmentation algorithm and the construction of reference model are shown in Fig. 3. On the basis of extracting ILM and RPE boundaries and unit conversion, take the average of unified boundaries as the normal retinal reference model, as shown the solid lines in Fig. 4. The dotted lines in the figure are the variation range based on reference model standard deviation.

Duan et al. [35] and Shen et al. [36] measured the macular thickness in healthy Chinese, and used OCT instruments are Stratus OCT (Carl Zeiss Meditec Inc., Jena, Germany) and Spectralis SD-OCT (Heidelberg Engineering, Heidelberg, Germany). Fast macular thickness scans were performed over macula within 6 mm in diameter, divided into 3 regions (central, inner, and outer, with a diameter of 1, 3 and 6 mm, respectively) and the specific metrical data are shown in Table 2, including fovea minimum, average thickness of central macula, inner and outer regions. The same metrical data obtained from the above reference model are also presented in Table 2. As the different data sources and thickness calculation methods, there are differences between the reference model and the numerical value in the literatures, but from the overall view, the thickness values of reference model are by a factor of about 1.25:1 larger than the values in literature [35] and 1:1.1 smaller than the values in literature [36]. The data showed that the reference model proposed in this paper is reliable.

**Table 2:** Normal macular measure thickness and reference model quantitative values ($\mu$m; mean ± standard deviations)

Thickness feature/source	Stratus OCT	Spectralis SD-OCT	Reference model
Fovea minimum	150.3 ± 18.1	215.4 ± 13.6	202.6 ± 12.5
Central (1-mm diameter)	176.4 ± 17.5	257.9 ± 19.2	233.6 ± 15.0
Inner region (3-mm diameter)	255.3 ± 14.9	339.2 ±14.6	313.7 ±15.5
Outer region (6-mm diameter)	237.7 ± 12.4	299.1 ± 14.3	286.5 ± 21.9

### Computerized quantification of retinal features

**Fig. 5:**
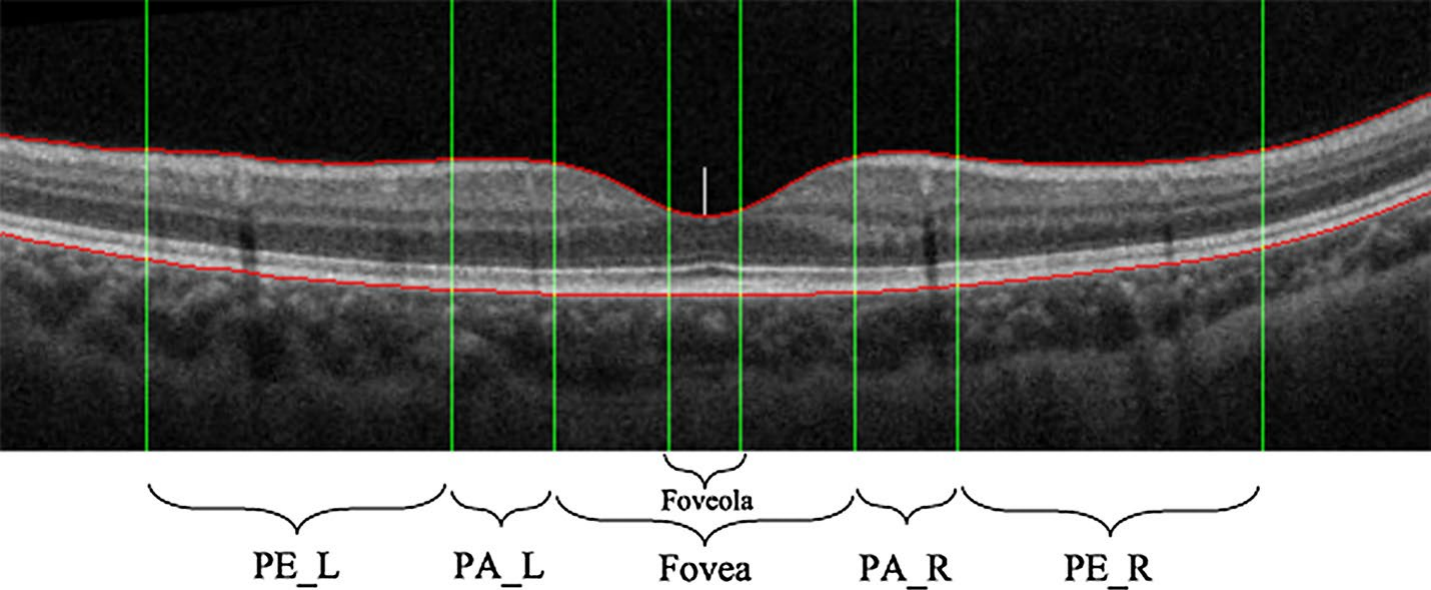
Schematic diagram of medical macular regional division. The *green lines* represent the medical macular regional division, including foveola, fovea, parafovea and perifovea. The *red lines* represent the *upper* and *lower* *boundaries* of retina. The *white short line* is the center position of foveola

In order to locate the position of lesion, the OCT image should be divided to regions before computation of quantification of retinal features. According to the anatomical definition of retina around macular regions, single OCT image is divided into five regions, namely, left perifovea (PE_L), left parafovea (PA_L), fovea (Fovea), right parafovea (PA_R), right perifovea (PE_R), as shown in Fig. 5.

The retinal morphological change is the indispensable basis for fundus anomaly judgment. When ophthalmologists analyze the retinal morphological change in OCT images, they focus on the retinal thickness, boundary smoothness and continuity. Retinal thickness is an important criterion for fundus disease diagnosis, and the location and severity of lesion could be determined by the change of thickness features. The size of retinal thickness data is related to the real condition of retinal tissue, instruments and the actual operation of the instruments. The data does not have the characteristics of feature invariant, therefore the definition of thickness ratio features can be more detailed to describe the geometrical features and thickness of retina. The standard deviation can reflect the degree of dispersion of a data set and the correlation coefficient is used to reflect the correlation between the variables. In this paper, the standard deviation and correlation coefficients were used to determine the relative degree and continuity between the target boundary and reference model. The gradient feature represents the change rate of parameters, and the curvature is a measure of the geometric uneven degree. So, the gradient and curvature features were used to express the smoothness of the boundary. In conclusion, this paper put forward the following three kinds of quantitative feature sets, as shown in Table 3, in order to realize the computerized quantification of medical features that ophthalmologists focus on.

**Table 3:** Computerized quantification of retinal features (*Regions* = [PE_L, PA_L, Fovea, PA_R, PE_R])

Ophthalmologists focus features	Computer quantitative features	Feature type
Retinal thickness	Thickness feature ($T_{Foveola}$,$T_{Regions}$ ),	Geometric features
	Thickness ratio feature ($TR_{Regions}$)	
Boundary continuity	Standard deviation ($\sigma _{ILM}$, $\sigma _{RPE}$),	Morphological features
	Correlation coefficient ($r_{ILM}$, $r_{RPE}$)	
Boundary smoothness	Gradient ($\nabla Y_{ILM}$, $\nabla Y_{RPE}$),	Morphological features
	Curvature ($K_{ILM}$, $K_{RPE}$)	

The thickness features are defined as the average retinal thickness of different regions in Fig. 4, and the definition of thickness ratio feature is $TR_{Regions} = T_{Regions}/ T_{Foveola}$. The morphological features are defined as follows:

Suppose *Y*(*i*) is the discrete data of ILM/RPE curve, $i = 1,2,3\ldots ,W$, *W* is the number of image horizontal pixels, namely, the width of image. Definition of *L* for the horizontal length of one area in *Regions*, $0<L\le W$.

Definition of regional mean curvature:

1 $$\begin{aligned} K =\frac{\sum _{i=1}^L K(i)}{L},\quad K(i)=\frac{|Y''(i)|}{\sqrt{(1+Y'(i)^2)^3}} \end{aligned}$$

where $$Y'(i)=Y(i+1)-Y(i)$$, $$Y''(i)=Y'(i+1)-Y'(i)=Y(i+2)-Y(i)$$.

Definition of regional mean horizontal gradient:

2 $$\begin{aligned} \nabla Y=\frac{\sum _{i=1}^L \nabla Y(i)}{L}, \quad\nabla Y(i)= \frac{\alpha Y}{\alpha x}(i)=Y(i+1)-Y(i) \end{aligned}$$

Suppose $Y_{ref} (i)$ is the discrete data of reference model ILM/RPE curve, and $Y_{tar} (i)$ is the discrete data of target ILM/RPE curve.

Definition of correlation coefficient between target curve and the reference model in a certain area:

3 $$\begin{aligned} r=\frac{\sum _{i=1}^L (Y_{ref} (i)-\overline{Y}_{ref})(Y_{tar} (i)-\overline{Y}_{tar})}{\sqrt{\sum _{i=1}^L (Y_{ref} (i)-\overline{Y}_{ref})^2 \cdot \sum _{i=1}^L (Y_{tar} (i)-\overline{Y}_{tar})^2}} \end{aligned}$$

where $\overline{Y}_{ref}$ is the regional mean of $Y_{ref} (i)$, and so is $\overline{Y}_{tar}$.

Definition of standard deviation between target curve and the reference model in a certain area:

4 $$\begin{aligned} \sigma = \sqrt{\frac{1}{L} \sum _{i=1}^L (D(i)-\overline{D})^2}) \end{aligned}$$

where $D(i)= Y_{tar} (i)-Y_{ref} (i)$, $\overline{D}$ is the regional mean of *D*(*i*).

Formulas (1)–(4) are unified expressions. According to the specific condition of ILM and RPE, these definitions can be respectively represented as $K_{ILM}$, $K_{RPE}$, $\nabla Y_{ILM}$, $\nabla Y_{RPE}$, $r_{ILM}$, $r_{RPE}$, $\sigma _{ILM}$, $\sigma _{RPE}$.

According to the 100 normal OCT images which are used to construct the reference model, after statistical analysis and calculation, the features data of retinal thickness and retinal thickness ratio are shown in Tables 4 and 5.

**Table 4:** Retinal thickness feature data ($\mu$m; mean ± standard deviations)

Computer quantitative feature names	Ref model value
Foveola average thickness $T_{Foveola}$	206.8 ± 13.2
Fovea average thickness $T_{Fovea}$	253.7 ± 14.1
Left parafovea average thickness $T_{PA\_L}$	320.2 ± 15.9
Right parafovea average thickness $T_{PA\_R}$	319.3 ± 15.6
Left perifovea average thickness $T_{PE\_L}$	296.1 ± 20.8
Right perifovea average thickness $T_{PE\_R}$	295.5 ± 20.1

**Table 5:** Retinal thickness ratio feature data

Computer quantitative feature names	Definition	Ref model value
Fovea thickness ratio $TR_{Fovea}$	$T_{Fovea}/T_{Foveola}$	1.22 ± 0.04
Left parafovea thickness ratio $TR_{PA\_L}$	$T_{PA\_L}/T_{Foveola}$	1.55 ± 0.10
Right parafovea thickness ratio $TR_{PA\_R}$	$T_{PA\_R}/T_{Foveola}$	1.54 ± 0.10
Left perifovea thickness ratio $TR_{PE\_L}$	$T_{PE\_L}/T_{Foveola}$	1.43 ± 0.13
Right perifovea thickness ratio $TR_{PE\_R}$	$T_{PE\_R}/T_{Foveola}$	1.43 ± 0.13

### Retinal abnormal grading decision-making

The retinal abnormal degree and lesion location could be identified by the diversity factor between the extracted ILM, RPE boundaries and reference model based on the feature parameters in Tables 3, 4 and 5 and the retinal feature value range around normal macula. Two kind of grading standard for evaluation of the abnormal degree, according to the different feature types, were proposed in this paper.

**Fig. 6:**
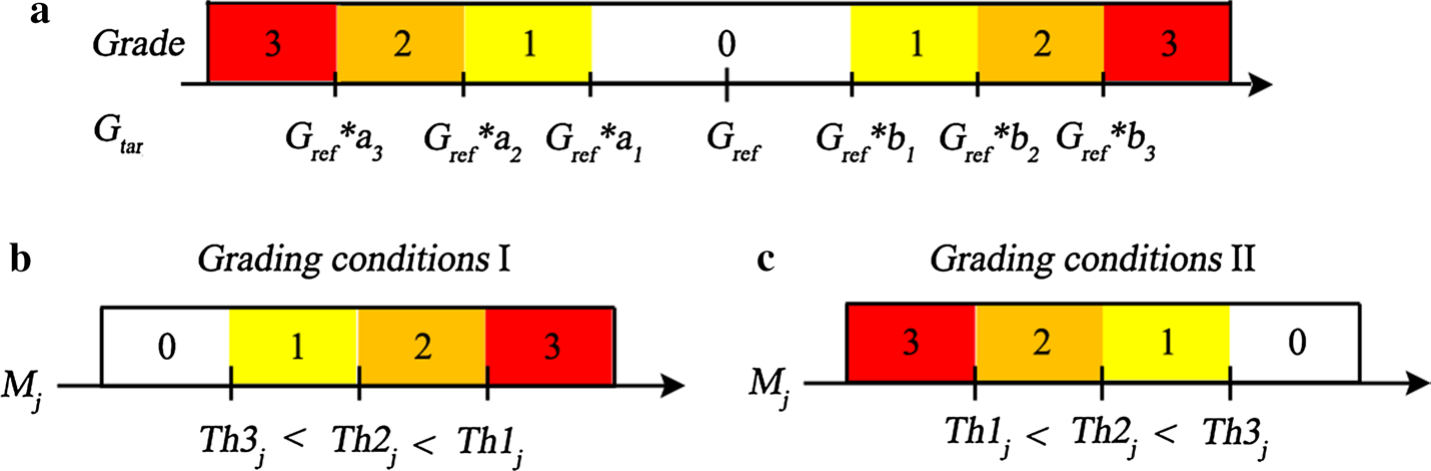
Diagrams of retinal abnormal grading decision-making. **a** The grading diagram for geometric features, and the abnormal grade is determined by the comparison result between the geometric features value of target image $G_{tar}$ and the geometric features value of reference model $G_{ref}$. **b**, **c** The diagrams for morphological features according to the different threshold sets

(1) Geometric features (thickness and thickness ratio) grading standard can be seen in Table 6 and Fig. 6a, where $G_{tar}$ represents the geometric features value of target image to be graded, $G_{ref}$ represents the geometric features value of reference model. The normal or not of the target image geometric features is judged by the deviation from reference model. Either large or small are abnormal, and the degree of abnormality is judged by the deviation. The deviation scope was determined by grading ratio factor and reference model based on the practical diagnosis experience of ophthalmologists. This paper set up the grading ratio factor $a_{1}$ = 0.8, $a_{2}$ = 0.6, $a_{3}$ = 0.5, $b_{1}$ = 1.2, $b_{2}$ = 1.4, $b_{3}$ = 1.5.

**Fig. 7:**
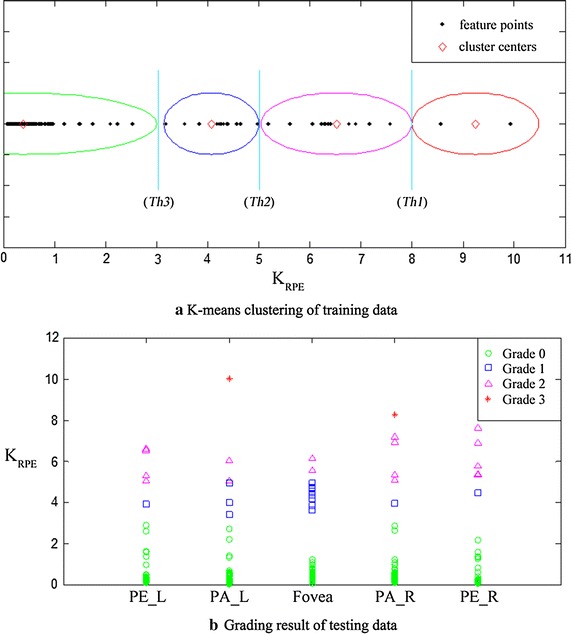
Diagrams of feature data processing. **a** The feature values from 50 training images. These feature values were divided into four groups and three thresholds were acquired. **b** The grading results of testing data by the acquired thresholds

**Table 6:** The grading decision-making of geometric features

Grading conditions	Grade	Deformation
$G_{ref}* b_{3}\le G_{tar}$	3	Serious
$G_{tar}\le G_{ref}* a_{3}$	3	Serious
$G_{ref}* b_{2}\le G_{tar}< G_{ref}* b_{3}$	2	Moderate
$G_{ref}* a_{3} < G_{tar}\le G_{ref}* a_{2}$	2	Moderate
$G_{ref}* b_{1}\le G_{tar}< G_{ref}* b_{2}$	1	Small
$G_{ref}* a_{2} < G_{tar}\le G_{ref}* a_{1}$	1	Small
$G_{ref}* a_{1}< G_{tar}< G_{ref}* b_{1}$	0	Normal

(2) Morphological features grading standard shows in (Table 7; Fig. 6b, c), where $M_j$ represents the value of feature $j, j=[\sigma _{ILM},r_{ILM},\nabla Y_{ILM},K_{ILM},\sigma _{RPE},r_{RPE},\nabla Y_{RPE},K_{RPE}]$. $Th1_j$ , $Th2_j$, $Th3_j$ represent the corresponding to three thresholds of feature *j*, and they are obtained by K-means clustering algorithm according to 50 training images with different abnormal severity selected by ophthalmologists. The basic clustering method divides the 50 data of each feature in each region into 4 groups. Where, the data of the region with similar feature, according to the symmetry, can be aggregated and clustered, such as PE_L and PE_R. Some features have the similar feature distribution in all five regions, and all data can be aggregated, such as $K_{RPE}$. The number of clustering data involved in this paper is 50, 100, 250. Figure 7 shows the data processing diagram of feature $K_{RPE}$. Image a in Fig. 7 displays the obtained 250 $K_{RPE}$ value points from 50 training images. These feature values were divided into four groups by clustering algorithm and the three thresholds were acquired. When we obtain new testing images, then we make the abnormal grading decision by the acquired thresholds, as shown in Fig. 7b. The standard is divided into two kinds of grading conditions according to the different threshold sets. Table 8 shows the reference thresholds for morphological features grading, where ($\nabla Y_{ILM}$ (P) | PE_L) represents the ILM positive gradient feature in left perifovea (PE_L). Through the observation of normal retinal reference model, we can find that on the left side of foveola, ILM and RPE show negative gradient trend, and on the right side of foveola, ILM and RPE show positive gradient trend. So in this paper, the morphological abnormalities were determined by the positive gradient value ($\nabla Y$(P) | PA_L), ($\nabla Y$(P) | PE_L) on left parafovea, left perifovea and negative gradient value ($\nabla Y$(N) | PA_R), ($\nabla Y$(N) | PE_R) on right parafovea, right perifovea. In the region of fovea, the subtraction between positive gradient values and negative gradient values was used to determine the morphological abnormalities, such as ($\nabla Y |$ Fovea) = ($\nabla Y$(P) | Fovea)-($\nabla Y$(N) | Fovea).

**Table 7:** The grading decision-making of morphological features

Grading conditions I $Th3_{j}< Th2_{j}< Th1_{j}$	Grading conditions II $Th1_{j}<Th2_{j}< Th3_{j}$	Grade	Deformation
$Th1_{j}\le M_{j}$	$M_{j}\le Th1_{j}$	3	Serious
$Th2_{j}\le M_{j} < Th1_{j}$	$Th1_{j}<M_{j}\le Th2_{j}$	2	Moderate
$Th3_{j}\le M_{j} < Th3_{j}$	$Th2_{j}<M_{j}\le Th3_{j}$	1	Small
$M_{j}< Th3_{j}$	$Th3_{j} < M_{j}$	0	Normal

**Table 8:** The reference threshold for morphological features grading

Morphological feature *j*	$Th1_{j}$	$Th2_{j}$	$Th3_{j}$	Grading conditions
$r_{ILM},r_{RPE}$	−0.9	−0.5	0	II
($\nabla Y_{ILM}$(P) | PA_L)($\nabla Y_{ILM}$(P) | PE_L)	20	8	3	I
($\nabla Y_{RPE}$(P) | PA_L)($\nabla Y_{RPE}$(P) | PE_L)				
($\nabla Y_{ILM}$(N) | PA_R)($\nabla Y_{ILM}$(N) | PE_R)	−20	−8	−3	II
($\nabla Y_{RPE}$(N) | PA_R)($\nabla Y_{RPE}$(N) | PE_R)				
($\nabla Y_{ILM} |$ Fovea)	70	50	30	I
($\nabla Y_{RPE} |$ Fovea)	35	25	10	I
$\sigma _{ILM},\sigma _{RPE}$	60	50	25	I
$K_{ILM},K_{RPE}$	8	5	3	I

## Results

**Fig. 8:**
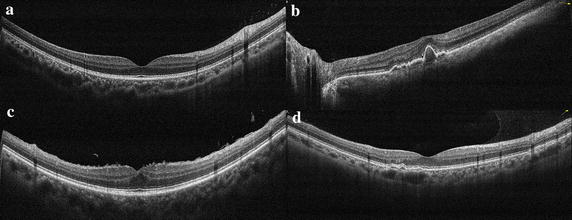
The original images shot by OCT instrument. **a** The normal retinal morphology, the fovea in **b** loses its normal form, and the RPE fluctuation is serious, the retinal surface of **c** appears wrinkles, the pigment epithelium uplifts on the *left side* of fovea in **d**

This paper showed the detailed analysis process by four retinal OCT images with different abnormal degrees. Figure 8 shows the original images shot by OCT instrument, where image a displays the normal retinal morphology, the fovea in image b loses its normal form, and the RPE fluctuation is serious, the retinal surface of image c appears wrinkles, the pigment epithelium uplifts on the left side of fovea in image d.

**Fig. 9:**
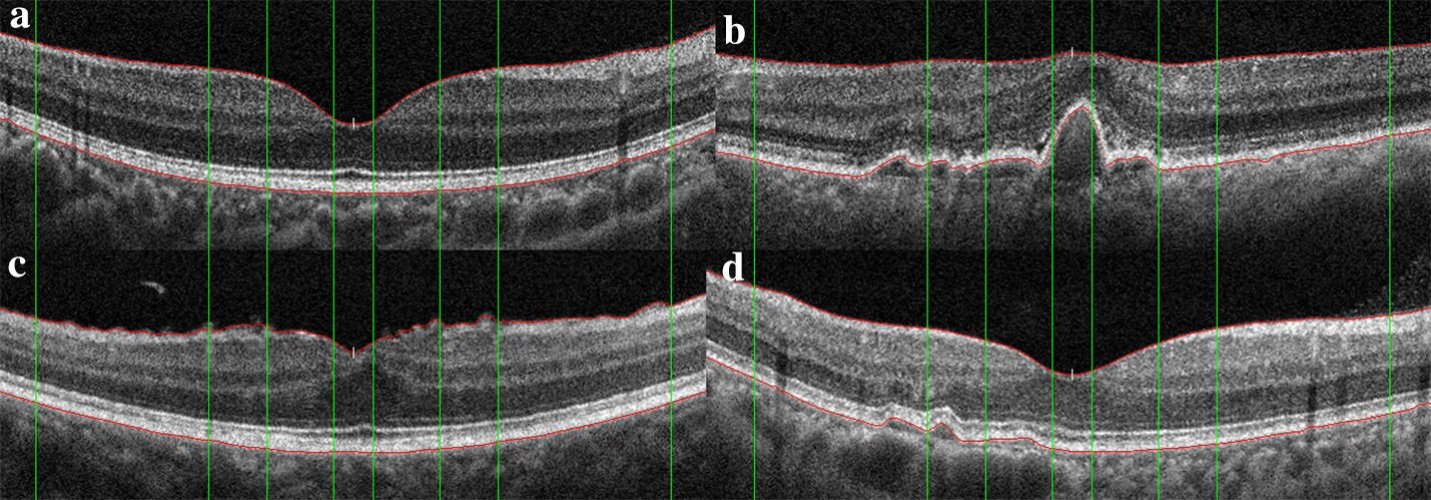
The results of retinal boundary extraction in OCT images. After the preprocessing of standardization and de-noising, the ILM and RPE boundaries are extracted based on intensity features, as shown in the *red lines*. The *green lines* represent the medical macular regional division boundaries. **a**–**d** are respectively corresponding to the four original images in Fig. 8

**Fig. 10:**
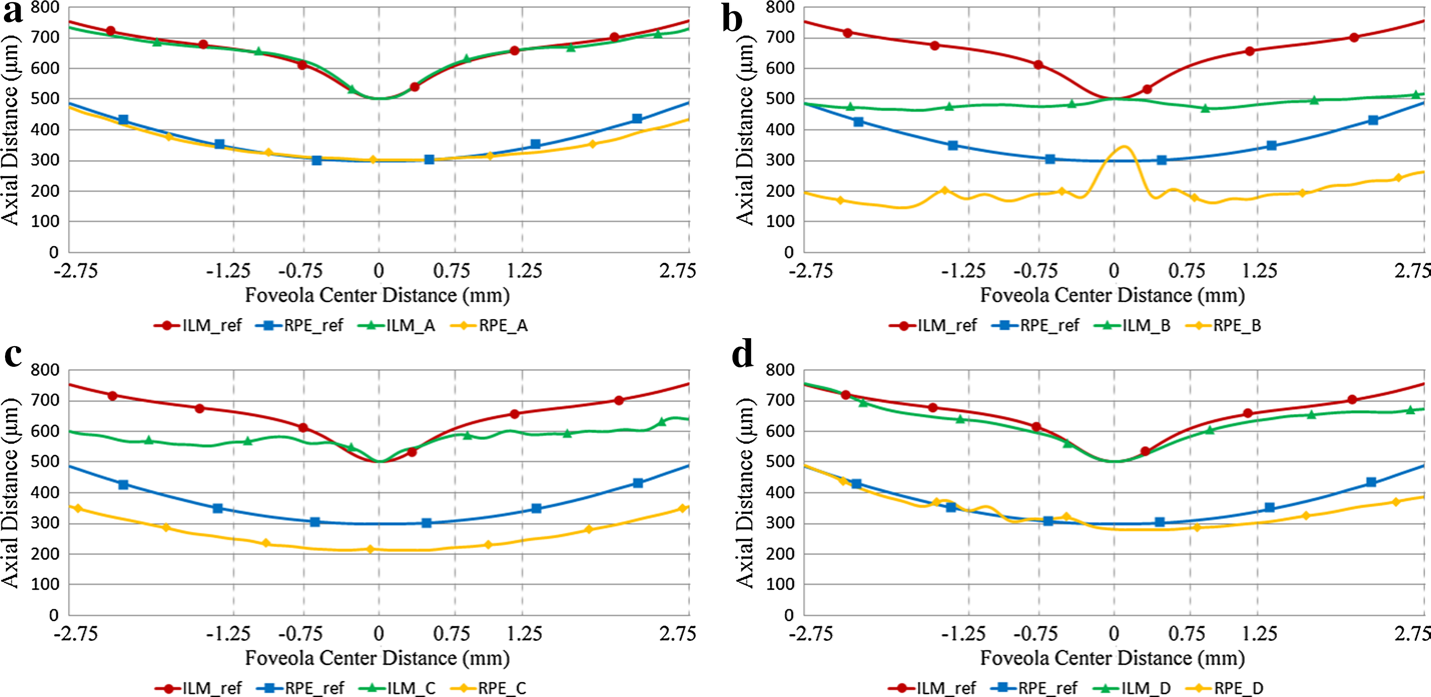
The ILM and RPE boundaries comparison with reference model. It is more intuitive to display the morphological difference by the retinal boundaries comparison between target images and reference model. **a**–**d** are respectively corresponding to the four original images in Fig. 8

After image standardization and de-noising, the macular region is in the center of new image and the whole retina is in a horizontal position. For each image in Fig. 8, ILM and RPE boundary extraction results are shown in Fig. 9. The red lines represent the ILM and RPE boundaries and the green lines represent the medical macular regional division boundaries. Figure 10 shows the curve comparison and morphological differences between reference model and extracted boundaries. The ILM and RPE boundaries in image a are very close to reference model and image b displays the large difference.

**Fig. 11:**
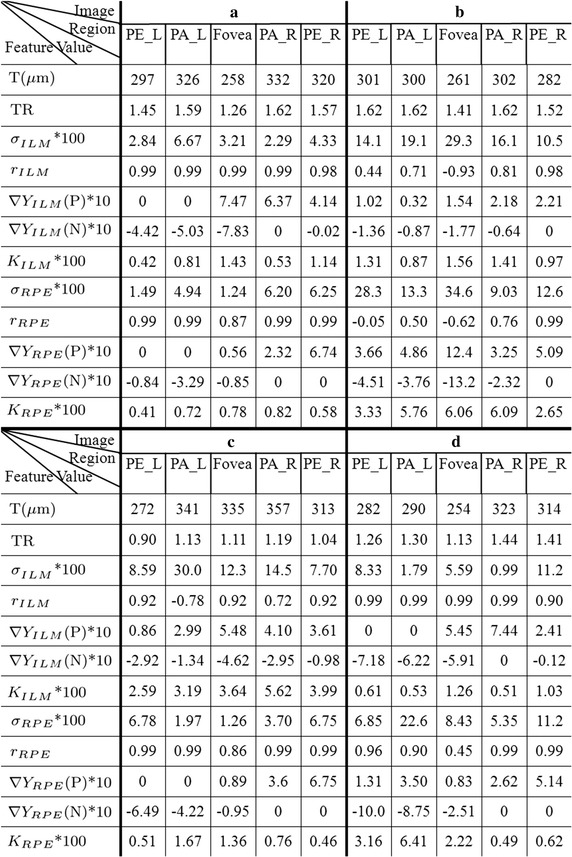
The features quantitative calculation results. The rough abnormities from the boundaries comparison are shown in Fig. 7 and more accurate judgment can be obtained by boundary structure quantification and data features. This figure shows the corresponding quantitative values in different regions and different features. **a**–**d** are respectively corresponding to the four original images in Fig. 8

The data features of five image regions, fovea (Fovea), left parafovea (PA_L), right parafovea (PA_R), left perifovea (PE_L), right perifovea (PE_R) are acquired based on ILM and RPE boundaries. The features quantitative calculation results of each standard RPE and ILM boundary membrane in Fig. 9 are showed in Fig. 11. $\nabla Y_{ILM}$(P) represents the positive gradient value of ILM and $\nabla Y_{ILM}$(N) represents the negative gradient value of ILM. RPE is in the same way.

**Fig. 12:**
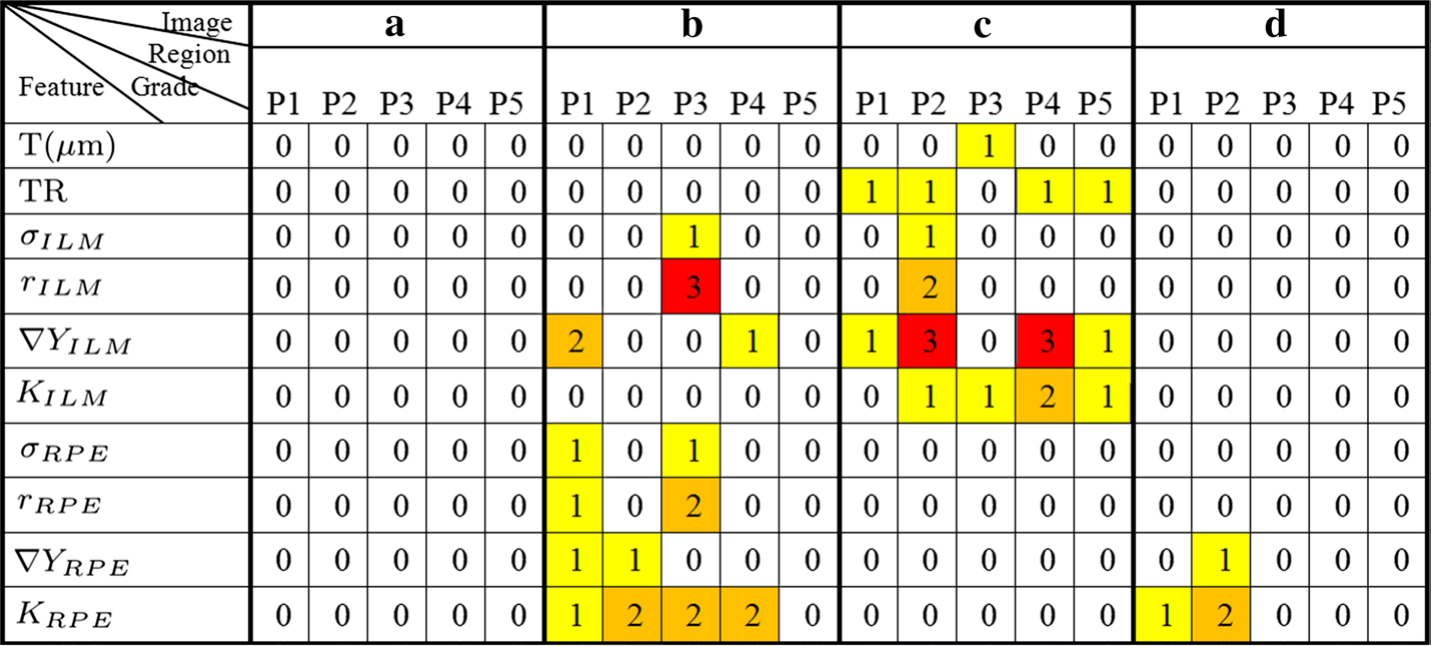
The grading results of quantitative data features. Through the quantitative data, combined with the reference model for abnormal grading, the regions and severity of abnormal can be more intuitive to display. As shown, **a** does not appear abnormal warning. While image **b** and **c** show larger abnormal grade and **d** shows abnormal in curvature and gradient features of RPE. P1, P2, P3, P4, P5 respectively correspond to PE_L, PA_L, Fovea, PA_R, PE_R regions

The data feature statistics results can be obtained by grading the quantitative data features. The corresponding evaluation grade of each image in different regions and different features can be get from Fig. 12, and the abnormal situation of retina could be judged by evaluation grade.

From Fig. 12 we can see that the image a does not contain abnormal warning and it is in line with the normal morphology. In image b, ILM and RPE have a higher level of abnormal grade in fovea area (P3), and RPE contains larger anomalies judged from the abnormal grade of RPE standard deviation and RPE mean curvature. In image c, the abnormal grade of thickness feature in fovea (P3) is level 1. Combined with the feature value, we can further judge that the fovea is thickened, resulting in the other areas (P1, P2, P4, P5) also appear anomalies in thickness ratio feature. In addition, the ILM gradient and the ILM mean curvature show a large abnormal grade, and the reason is that the ILM surface is uneven in image c. This kind of anomaly is mainly caused by epiretinal membrane. In image d, the RPE mean curvature appears abnormal numerical warning in left perifovea area (P1) and left parafovea area (P2), corresponding to the small uplift caused by drusen.

As mentioned before, this paper analyzed 300 retinal OCT images, including 200 images judged normal and 100 images judged abnormal. 100 normal images were used to construct the reference model and 50 abnormal images were utilized for the grading thresholds. So the remaining 100 normal images and 50 abnormal images were utilized to validate that this method was scientifically valid.

**Fig. 13:**
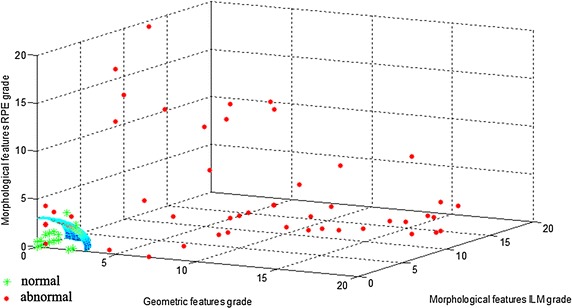
The scatter plot of testing images. The features in each image were grouped and converted into a three dimensional data point. The normal and abnormal images labeled by ophthalmologist were marked for different *scatter points*. The testing images were judged normal or abnormal based on the 1/8 spherical surface in our algorithm. The points in the inner space of spherical surface were judged normal and the points in the outer space were judged abnormal. It can be seen from the image that the abnormal judgment method presented in this paper is basically consistent with the results of ophthalmologists

There are 10 features for grading in Fig. 12 and we split these features into three groups, namely Geometric features, Morphological features ILM and Morphological features RPE. Then we summed the feature grade in each group and the features in each image were converted into a three dimensional data point. For example, image a–d can be represented as data points (0,0,0), (0,7,14), (5,16,0), (0,0,4). All the testing images were converted into three dimensional data points and the normal and abnormal images labeled by ophthalmologist were marked for different scatter points, as shown in Fig. 13. The data points of normal images distributed in the vicinity of the point (0,0,0), and the higher degree of abnormal, the farther away from this point. In this paper, the testing images were judged normal or abnormal based on the 1/8 spherical surface in Fig. 13. The points in the inner space of spherical surface were judged normal and the points in the outer space were judged abnormal. In this paper, the radius of the spherical surface is 3.

The simulation results of 100 normal and 50 abnormal OCT images showed that the sensitivity was 0.94 and the specificity was 0.92 (where Specificity = TN/(FP + TN), Sensitivity = TP/(TP + FN), TN-true negative, TP-true positive, FN-false negative, FP-false positive). Correlation coefficient was the mainly affects feature of normal image erroneously detected as abnormal. Because the normal fundus also appeared the morphologies different with the reference model, and this kind of situation was also affected by the shooting angle. The main reason of abnormal image erroneously detected as normal was that some small macular edema appeared on the top of RPE. This kind of abnormality has not yet affected the lower edge of RPE, so it has not been detected, which is the direction of further research in this paper.

## Conclusions

With OCT data being generated in increasingly larger amounts and captured at higher resolution, there is a strong need for computer assisted analysis to support disease diagnosis and the automatic analysis of OCT images has remained an active field of research.

We presented an automatic analysis method of fundus retinal status, based on boundary extraction, morphological characterization, feature quantification and grade evaluation. First, a normal retinal reference model was presented on the base of extracted ILM and RPE boundaries. Then, this paper proposed a set of retinal features extraction and quantification methods, for providing numerical reference standard on inspection of retinal status by OCT images. After obtaining the retinal feature parameters, this paper judged the anomalies of target image combined with the reference model and formulated grading method, so as to conveniently and clearly judge the abnormal region and severity.

Different with former studies, like extraction of pathological area in known cases of illness or features extraction and classification of limited certain kinds of diseases, Our method would not limit the abnormal categories within the scope of certain kinds of known cases, because the retinal images are usually complex in practical diagnostic process. So the applicability and utility of our method is better in health screening and preliminary diagnosis of the retina.

This paper proposed a general framework for the realization of computerized medical diagnosis of retina and it is also a potentially valuable tool for remote diagnosis applications. In future research, we will refine the reference model and make use of more image features to realize the distinction between more ophthalmologies and identify different disease categories.

## Abbreviations

OCT: optical coherence tomography
BLS-GSM: Bayesian least squares–Gaussian scale mixture approaches
NL Means: non-local means
NCDF: nonlinear complex diffusion filter
CWDL: complex wavelet based dictionary learning methods
BM3D: block-matching and 3D filtering
CNR: contrast-to-noise ratio
TP: texture preservation
EP: edge preservation
ENL: equivalent number of looks
ILM: internal limiting membrane
RPE: retinal pigment epithelium
PA_L: left parafovea
PA_R: right parafovea
PE_L: left perifovea
PE_R: right perifovea
TR: thickness ratio
